# Immunization and Host Responses to *MB-1*, a Live Hatchery Vaccine against Infectious Bursal Disease

**DOI:** 10.3390/vaccines11081316

**Published:** 2023-08-02

**Authors:** Yossi Wein, Virginie Loeb, Aderajew Asmare, Saar Tal, Avner Finger, Aharon Friedman

**Affiliations:** 1 Department of Animal Sciences, R.H. Smith Faculty of Agriculture, Food and Environment, The Hebrew University of Jerusalem, P.O. Box 12, Rehovot 7610001, Israel; 2 Phibro Animal Health Corporation, P.O. Box 489, Beit Shemesh 99100, Israel; virginie.loeb@pahc.com (V.L.);

**Keywords:** *MB1*-vaccine, maternal antibodies, gumboro, infectious bursal disease (virus)

## Abstract

*MB-1* is an attenuated infectious bursal disease virus vaccine. Previously, we observed a temporal delay of vaccine virus replication in the bursae of chicks due to maternally derived antibodies (MDAs). The mechanism that allowed its survival despite MDA neutralization remained unclear. We hypothesized that after vaccination at 1 day of age (DOA), the *MB-1* virus penetrates and resides in local macrophages that are then distributed to lymphoid organs. Furthermore, *MB-1*’s ability to survive within macrophages ensures its survival during effective MDA protection. PCR analysis of lymphoid organs from chicks with MDA, vaccinated on 1 DOA, demonstrated that the *MB-1* virus was identified at low levels solely in the spleen pre-14 days of age. Fourteen days after vaccination, the virus was identified using PCR in the bursa, with viral levels increasing with time. The possible delay in viral colonization of the bursa was attributed to the presence of anti-*IBDV* capsid VP2 maternal IgA and IgY in the bursa interstitium. These indicate that during the period of high MDA levels, a small but viable *MB-1* viral reservoir was maintained in the spleen, which might have served to colonize the bursa after MDA levels declined. Thereafter, individual immunization of chicks against Gumboro disease was achieved.

## 1. Introduction

Gumboro disease, a highly contagious and environmentally resistant viral disease of young chicks, may lead to immune suppression and secondary infections accompanied by direct and/or indirect high mortality rates [1,2,3]. The disease’s causative agent is the infectious bursal disease virus (*IBDV*), a 60 nm non-enveloped double-stranded RNA virus with icosahedral symmetry from the genus *Avibirnavirus* in the *Birnaviridae* family, in which the main route of entry is oral and/or cloacal [1,2,3]. The *IBDV* viral protein 2 (VP2) serves as a viral capsid protein along with VP3 and is one of the five major viral proteins of *IBDV*. As the unique viral protein exposed on the surface, VP2 plays a major role in the production of neutralizing antibodies [1,2,3,4].

Due to its high tropism to immature B-lymphoblasts (surface IgM positive B lymphoblasts-sIgM^+^), *IBDV’s* main target organ is the bursa of *Fabricius* in 1–6-week-old chicks [2,5,6,7]. As other lymphatic organs, such as the spleen, thymus, and cecal tonsils, may also be targeted by *IBDV*; they are considered to be secondary target organs [1,2,3,8]. More recently, the ability of *IBDV* to reside in macrophages has been demonstrated [1,2,5,7]. 

Interestingly, the *IBDV* disease phenotype varies with available immature B lymphoblast levels and is assumed to be age-dependent. This is reflected by early infection (up to 3 weeks of age), resulting in low mortality rates and permanent immunosuppression, and late infection (3–6 weeks of age), resulting in high mortality rates with transient immunosuppression [1,2,3]. To date, *IBDV* is a major threat to the poultry industry, and vaccination against Gumboro disease is the most successful strategy to protect poultry livestock from clinical disease [1,3,9].

Over the years, different vaccination strategies have been developed and practiced, such as: live attenuated, inactivated, sub-unit, vectored/recombinant, and immune complexes. Unsurprisingly, each one of these strategies possesses pros and cons, such as efficacy vs. safety, vaccine virus neutralization by MDA vs. impairment of maternal immunity, onset vs. duration of immunity, and finally, the ability to elicit both humoral and cellular immune response [10,11,12,13]. Nevertheless, live attenuated vaccination mimics infection by the field strain virus; it provides the best host protection to poultry by activating cellular and mucosal immune responses in addition to a humoral response [1,3,10,13,14]. As the virus infects young chicks, an efficient *IBDV* vaccine should provide early onset of immunization with continuous protection [1,3,10,11,12], especially for broilers, which have a very limited period to produce active immunity following the decay of maternally derived antibodies. Accordingly, the *MB-1* vaccine (Phibro Animal Health), designed for hatchery applications, is administered in ovo or to 1-day-old chicks via the subcutaneous route; it is a naked attenuated live vaccine. It is safe and efficient and registered in many countries, as previously published [10,11,12,14]. Previous observations showed that *MB-1* vaccination led to anti-*IBDV* antibody production as early as 23 days of age (DOA) [10,11,12]. 

Numerous studies have described the interaction between maternally derived antibodies (MDAs) and vaccines administered to neonates and hatchlings [15,16]. These studies indicate a dichotomous relationship: The vaccine may serve to deplete MDA and/or MDA block the vaccine and, thus, prevent protective immunity. Hence, evidence obtained by us and others showed that in individual *MB-1* immunized chicks, virus replication in the bursa was delayed according to the MDA levels [10,11,12]; this effect was not observed in SPF chicks and virus replication in the bursa was observed as early as 24 h post vaccination [10,11,17,18,19,20]. 

The possible interactions between *MB-1* vaccination, maternal antibodies, and virus tropism were studied herein according to the following hypothesis:

1. Once injected, the *MB-1* vaccine virus infects macrophages that then migrate via the bloodstream to B lymphoblast-rich organs; MDA impedes this spreading.
2. MDA levels vary between tissues and organs. Organs with low levels of MDA are more susceptible to infection, thereby keeping the vaccine virus viable.
3. The decay of systemic maternal protection offers the vaccine virus the opportunity to infect all previously unavailable tissues and organs. A protective immune response is elicited after critical mass infection, and the vaccine virus is eradicated.

## 2. Materials and Methods

### 2.1. Animals and Husbandry

Ross broiler’s chicks were reared in enclosed and environmentally controlled isolators (Herut Experimental Facility, Israel). Following a standard rearing protocol (Poultry Section, Ministry of Agriculture, Israel), the chicks were fed with a complete broilers ration, and water was provided *ad libitum* [21]. The photoperiod was 16:8 h of light and darkness, respectively. Rearing temperature was adapted to the age of the chicks and was kept identical in all groups.

### 2.2. Ethics Statement

All studies were performed under an Institutional Animal Care and Use Committee-approved protocol of the Ministry of Health—the Council for Animal Experiments in compliance with Animal Welfare Regulations (Approval no. IL-20-6-226).

### 2.3. MB-1 Vaccination

On 1 DOA, 161 chicks were randomly tagged and divided into 2 groups (N = 113 per vaccinated group, N = 48 for naïve control group). Vaccinated group chicks were vaccinated subcutaneously (S.C.) with one dose of the *MB-1* vaccine according to the manufacturer’s (Phibro Animal Health, Israel) instructions. Control group chicks (naïve) were injected with *MB-1* and an identical volume of vaccine-diluent buffer Lactated Ringer’s solution (LRS, Teva Medical LTD, Ashdod, Israel).

### 2.4. Organs and Blood Collection

Overall, 18 chicks served to determine the time course of seroconversion (the same chicks at each time point), and 125 chicks served to determine the time course of viral load, antibody levels, and immune complexes within organs (different chicks were used at each time point due to sacrifice for organ sampling). Specifically, at eight predetermined time points (4, 8, 11, 14, 18, 23, 28, 32, and 36 DOA), ten chicks from the vaccinated group and three chicks from the naïve group (at day 36, fifteen chicks from the vaccinated group and six chicks from naïve group) were sacrificed for bursa, spleen, thymus, and bile fluid collection.

Determination of the necessary chick number for this research was performed based on our previously published observations [10,11,12,20] and following consultation with a statistician (Dr. Yotva Lavi), to allow sufficient statistical power along with minimal chicks usage. Accordingly, preliminary data were used to determine the number of chicks needed to be included in the trial in order to receive significant results (α = 0.05), with a study power of at least 0.8. The preliminary data included similar two-way ANOVA designs that we used in the past. 

At the same time points (with an additional time point at 1 DO, before vaccination), blood was collected from nine vaccinated chicks and nine naive chicks. Within the isolator, chicks were randomly selected, manually restrained, and 1.5 mL of blood was drawn via venipuncture of the *vena cutanea ulnaris* and divided into two tubes: the first tube containing TRI Reagent^®^-BD (Molecular Research Center Inc., Cincinnati, OH, USA) and designated for viral RNA extraction and further viral load quantification; the second tube, Vacuette^®^ Z Serum Sep Clot Activator (Greiner Bio-one, KremsmÜnster, Austria) was designated for serum preparation and serology via ELISA.

For FACS (Fluorescence Activated Cell Sorting) and confocal microscopy analysis at 4 DOA, an additional 9 vaccinated and 9 naïve chicks were sacrificed, and spleens were sampled into a tube containing RPMI 1640 medium with HEPES 25 mM and L-Glutamine (Biological Industries, Kibbutz Beit Haemek, Israel). 

### 2.5. RNA Extraction and PCR Analysis

Each chick’s sampled bursa, spleen, and thymus were homogenized separately using a polytron PT2500E (Kinematica, Malters, Switzerland). The extract was then centrifuged at 400× *g* for 10 min at 4 °C, and RNA was extracted from the supernatant using a QIAamp viral RNA mini kit (Qiagen, Hilden, Germany) according to the manufacturer’s instructions. Additionally, RNA was extracted from chicken PBL using TRI Reagent™ (Molecular Research Center Inc., Cincinnati, OH, USA) according to the manufacturer’s instructions. Thereafter, 300 nanograms of RNA from each sample were reverse transcribed, and cDNA was amplified using qScript XLT 1-Step RT-qPCR ToughMix (QuantaBio, Beverly, MA, USA) and specific primers and probes for *IBDV-MB-1* segment A genes. Primer and probe sequences were designed using Oligo primer analysis software (Molecular Biology Insights, Inc., Colorado Springs, CO, USA) according to a GeneBank published sequence (DQ927040.1) and Techera et al., 2019; primers and probes were as follows:
**Primer****Sequence**IBDV_F825′-CAAGATCAAACCCAACAGATTG-3′IBDV_R2535′-CTCTGACCTGAGAGTGTGCTTCTC-3′IBDV_P175FAM-ACGGAGCCTTCTGATGCCAACAAC-NFQ

Primer pairs and probes were calibrated to determine the optimal reaction temperature and cDNA concentration. Expression levels of examined genes were determined via qPCR using a C1000 Thermal Cycler, and the results were analyzed using Bio-Rad’s CFX manager™ software (http://www.biorad.com/webroot/web/pdf/lsr/literature/10021337.pdf), (accessed on 1 June 2023) (Bio-Rad, Hercules CA, USA). Viral load was expressed by comparing Cq values to a standard curve produced from an *MB-1* vaccine with a known titer. 

### 2.6. Determination of Serum Anti-IBDV IgY

Anti-*IBDV* IgY levels were determined in serum samples using the IBD-XR Ab EISA Test (IDEXX Laboratories, Inc., Westbrook, ME, USA), according to the manufacturer’s instructions.

### 2.7. Determination of Organs and Bile Anti-IBDV IgY and IgA Antibody Levels

Levels of anti-*IBDV* IgY and IgA antibodies within organs and bile fluids were determined via quantitative and qualitative indirect ELISA, respectively.

Briefly, for both assays, recombinant *IBDV-VP2* (Phibro Animal Health, Israel) was coated onto ELISA plates (Nunc, Thermo Fischer Scientific Inc., Rockford, IL, USA) while diluted in a carbonate–bicarbonate buffer. Coated plates were incubated in a humidified chamber at 4 °C overnight and then blocked using 0.5% skim milk (BD, Difco, Sparks, MD, USA) in PBS. Following extensive plate washing (wash solution, Kirkegaard and Perry Laboratories, Gaithersburg, MD, USA), diluted samples of the supernatant from the organ extracts, previously centrifuged at 6000× *g* for 10 min at room temperature, were added. In parallel, a standard curve of in-house affinity-purified chicken anti-*IBDV*-VP2 IgY was applied to make the IgY assay quantitative. The plates were then placed again in a humidified chamber at 4 °C overnight. Detection was performed using HRP conjugated polyclonal goat anti-chicken IgY-Fc specific or polyclonal goat anti-chicken IgA α chain specific (Bethyl Laboratories, Montgomery, TX, USA), TMB substrate (Kirkegaard and Perry Laboratories, Gaithersburg, MD, USA) and 450 nm Stop Solution for TMB substrate (Abcam, Cambridge, UK). Optical absorbance was determined at 450 nm using a Bio Tek microplate reader (Bio Tek, Winooski, VT, USA). Anti-*IBDV* IgY levels were quantified by comparing absorbance values to the standard curve, while anti-*IBDV* IgA levels were determined qualitatively. 

### 2.8. Determination of Organ IgY-IBDV Immune Complexes by ELISA

Briefly, ELISA plates were coated with 2.5 µg/mL monoclonal mouse anti-*IBDV*-VP2 IgG (Abnova, Taipei City, Taiwan) diluted in a carbonate–bicarbonate buffer. Coated plates were incubated at 4 °C overnight and then washed and blocked, as described above. Organ samples prepared as described above were added, and subsequently, plates were incubated overnight in a humidified chamber at 4 °C. The presence of IgY-*IBDV* immune complexes was detected using HRP conjugated polyclonal goat anti-chicken IgY-FC specific (Bethyl Laboratories, Montgomery, TX, USA). For specificity testing, plates were incubated with either recombinant *IBDV-VP2* (50 μg/mL) (Phibro Animal Health, Israel) or naïve samples.

### 2.9. IBDV-MB-1 Localization within Splenocytes by Fluorescence Activated Cell Sorting (FACS) Analysis

Fresh spleens harvested from 9 vaccinated and 9 naïve chicks were placed into tubes containing RPMI 1640 medium with HEPES 25 mM and L-Glutamine (Biological Industries, Kibbutz Beit Haemek, Israel) and crushed using the flat end of a 5 mL plunger and cell culture metal mesh. The single-cell suspension was then transferred through a 40 μm nylon Falcon™ cell strainer (Thermo-Fisher Scientific, NY, USA) into fresh 50 mL conical tubes. Cell vitality was evaluated to be ~95% after staining with 0.5% trypan blue (Biological Industries, Kibbutz Beit Haemek, Israel). Then, 3 samples per treatment were pooled together, constituting 3 pooled samples each for both treatments. Pooling was performed due to the chick spleen’s small size and to ensure sufficient cell yielding for FACS analysis. Data obtained from preliminary experiments suggested that the pooling of 3 spleens would ensure sufficient cell yields with minimal variability between samples. Splenocytes were fixed for 10 min using 1.6% formaldehyde in PBS (Sigma-Aldrich, Saint Louis, MO, USA) and subsequently permeabilized for 15 min using 0.2% Tween 20 (Sigma-Aldrich, Saint Louis, MO, USA) in PBS.

Cells (1 × 10^6^ cells/tube) were double stained for viral detection and the determination of splenocyte subpopulations: B lymphocytes, immature B lymphoblasts, and macrophages. *MB-1 IBDV* viral detection was performed using monoclonal mouse anti-*IBDV*-VP2 IgG (Abnova, Taipei City, Taiwan), conjugated to AF488 using Lynx Rapid Plus DyLight^®^488 antibody conjugation kit (Bio-Rad, Hercules CA, USA), according to the manufacturer’s instructions. Splenocyte sub-populations were determined using AF647 conjugated mouse monoclonal antibodies recognizing chicken: BU-1 for B lymphocytes, IgM for immature B lymphoblasts, and KUL1 for macrophages (SouthernBiotech, AL, USA). Preliminary experiments showed no effect on cell population dispersion following the fixation and permeabilization procedures. AF488 IgG and AF647 IgG isotype controls were used as internal negative controls (SouthernBiotech, AL, USA).

Briefly, AF488 conjugated anti-*IBDV*-VP2 IgG and AF647 conjugated anti-BU-1 or IgM or KUL1 were added to each splenocyte pool in 1% BSA (Sigma-Aldrich, Saint Louis, MO, USA) in PBS and incubated at 4 °C over-night. The cells were then thoroughly washed and filtered, using a 40 μm nylon Falcon™ cell strainer (Thermo-Fisher Scientific, NY, USA), into Falcon™ polystyrene FACS tubes (Becton Dickinson, NJ, USA). A tube with the isotype control served as a negative control. In total, 10,000 cells of each pool were counted, and analysis was performed using BD Accuri C6 plus Cell Analyzer front, side scatters, FL-1, and FL-4 characteristics. 

### 2.10. Fluorescence Confocal Microscopy Assay

Following staining and FACS assay, cells from each pool were smeared over positively charged microscope slides and counterstained using Fluoroshield™ with DAPI (Sigma-Aldrich, Saint Louis, MO, USA). Pictures were taken using an SP8 Lightning Confocal microscope (Leica, Germany).

### 2.11. Statistical Analysis

Statistical analyses were performed using JMP^®^ software (SAS^®^ Institute Inc., Cary NC, USA). For data with equal variances, main effects were analyzed using 1-way or 2-way Anova, following Tukey HSD for multiple comparisons or Welch–Student t for paired comparisons. For data with unequal variances, main effects were analyzed using nonparametric Wilcoxon and each paired test was used for multiple comparisons.

FACS data were analyzed by FlowJo software (FlowJo, LLC data analysis software, Ashland, OR, USA), and statistical analysis was performed using Kolmogorov–Smirnov test (K–S test): a nonparametric test of the equality of continuous, one-dimensional probability distributions, used to compare two or more samples (naïve Vs vaccinated pools). 

## 3. Results

Understanding the mode of action of *MB-1* vaccine immunization initially required the establishment of the time course of maternally derived serum anti-*IBDV* IgY. Characterization of the anti-*IBDV* antibody response (maternal and *de novo*) was followed and compared for 36 days in both vaccinated (*MB-1* s.c. at 1 DOA) and naïve broiler chicks. 

**Figure 1:**
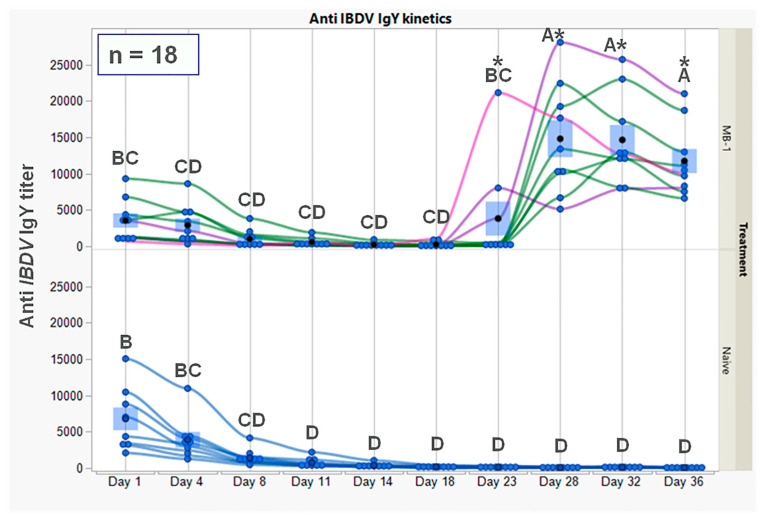
Serum anti-*IBDV* IgY kinetics in vaccinated and naïve broiler chicks, following vaccination at 1 DOA with *MB-1* vaccine. Antibody titers were obtained using the IDEXX IBD-XR Ab ELISA test. Each blue point represents an individual chick at a specific time point. For vaccinated chicks only, green lines represent chicks with relatively high anti-*IBDV* MDA, while pink and purple lines represent chicks with relatively low anti-*IBDV* MDA (each line stands for repeated measures of the same chick). For each time point and treatment, blue bars represent the mean ± SEM of 9 individual chick measurements. A two-way ANOVA model in a repeated measurements design was used to determine the significance of differences between group mean values; groups or days headed by different letter combinations are significantly different (*p* < 0.05). For days 23–36, due to heterogeneity of variance between groups within days, the significance of differences between groups’ mean values was determined via Welch-*t*-test (*p* < 0.05); differences were marked using asterisks *.

The results presented in Figure 1 show high and similar titers of maternally derived anti-*IBDV* IgY in both vaccinated (mean: 4766 ± 1356) and naïve (mean:7743 ± 2032) groups at 1 DOA. This was followed by a similar decline in IgY levels, with undetectable levels at 18 DOA (436 ± 125 and 217 ± 59 for the vaccinated and naive groups, respectively). Thereafter, a significant increase in antibody levels occurred only in the vaccinated group from 18–23 DOA (see figure legend for analysis), this being a result of *de novo* antibody production. The antibody levels then remained relatively stable up to 36 DOA. Interestingly, during seroconversion, a high degree of antibody variability was observed between individual vaccinated chicks; some chicks underwent seroconversion at 18–23 DOA (4456 ± 2423) while others at 23–28 DOA (18,486 ± 2423). It appeared that most chicks with initial low MDA (2302 ± 929) went through early seroconversion, while chicks with initial high MDA (8040 ± 1925) went through later seroconversion at different scores; *p*-value = 0.0363.

The time course of anti-*IBDV* IgY in serum, as described above, supports our hypothesis on the survival of the *MB-1* vaccine even in the presence of neutralizing MDA. This observation suggested the following mode of action: The effectiveness of MDA prevented *MB-1’s* ability to infect its primary target site, the bursa of *Fabricius*, and *MB-1’s* ability to survive within macrophages ensures its survival during effective MDA protection; once MDA protection is diminished, the *MB-1* virus manages to infect naïve B cells. To support this mode of action, we quantified and compared *MB-1* viral loads in the bursa, spleen, thymus, and blood using qPCR.

**Figure 2:**
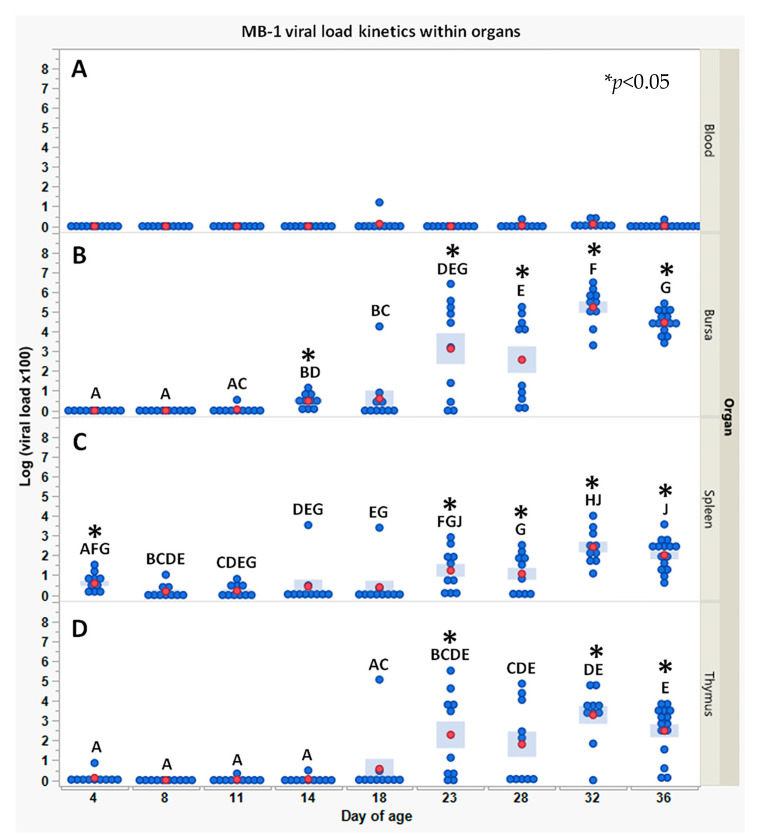
*MB-1* viral load kinetics within the blood (**A**), bursa (**B**), spleen (**C**), and thymus (**D**) in vaccinated chicks, following 1 DOA vaccination with *MB-1* vaccine; in all naïve chicks, viral load was 0 (data not shown). Viral loads were obtained using qPCR and translating Cq values using a known *MB-1* standard (as described in the Section 2). Each blue point represents an individual chick at a specific time point. For each time point and organ, red points and blue bars represent the mean ± SEM of 10 individual chick measurements (15 measurements at 36 DOA). Due to heterogeneity of variance between days, the significance of differences between days’ mean values was determined via non-parametric Wilcoxon each paired test; days headed by different letter combinations are significantly different (*p* < 0.05). The significance of differences between groups within days’ mean values was determined via Welch-*t*-test (*p* < 0.05); differences were marked using asterisks *.

As expected, samples from naïve chicks in all tested organs and at all time points showed zero viral loads (data not shown). In the immunized chicks, the viral load within the blood (Figure 2A) was low to absent at all tested time points, with values close to the limit of detection. In the bursa of *Fabricius* (Figure 2B), the *MB-1* viral load became detectable on 14 and 18 DOA; these levels, as shown in the figure, were significantly above the limit of detection (the viral load of one chick on 18 DOA was particularly high). Viral loads significantly increased in both chick numbers and levels from 18 DOA till they peaked at 32 DOA (see figure for analysis); this was followed by a minor, yet significant, decrease at 36 DOA (the significance of the differences between time points is given in the figure). High variability between individuals was observed at 23–28 DOA. Interestingly, viral loads were detectable in the spleen of single chicks as early as 4 DOA. These minimal viral loads remained stable till 18 DOA (the viral load of one chick at 14 DOA and one at 18 DOA, respectively, was particularly high). Thereafter, a moderate and significant increase in viral load was observed in most birds between 23 and 28 DOA till it peaked at 32–36 DOA, with all birds responding. Viral load levels observed in the spleen were several logs lower than those observed in the bursa. A similar pattern to the viral load in the bursa was observed with the viral load in the thymus (Figure 2D): very low initial loads close to the detection limit were observed between 4 and 18 DOA (except for one chick at 18 DOA). Starting from 23 DOA, viral loads increased significantly in most chicks and remained stable till 36 DOA; A high degree of variability between individual chicks was observed between 23 and 36 DOA. Additionally, as in the spleen, peak viral load levels were several logs lower than those observed in the bursa. 

A possible interaction between the host’s immune system (antibodies) and the vaccine virus strain (*MB-1 IBDV)* in the vicinity of its primary target organ (the bursa of *Fabricius*) may shed light on the dynamics of the immunization process. To investigate this interaction, a novel in-house developed ELISA was performed to locate IgY-*IBDV* (VP2) immune complexes in the bursa interstitial fluid.

**Figure 3:**
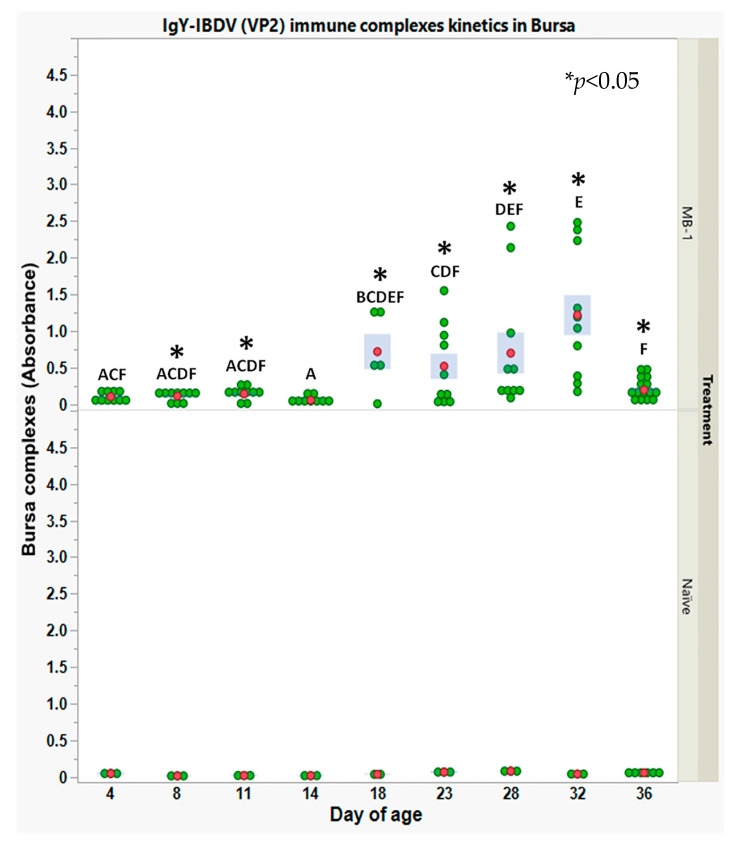
IgY-*IBDV* immune complexes kinetics within bursa interstitial fluids in naïve and vaccinated chicks, following 1 DOA vaccination with *MB-1* vaccine. Levels of immune complexes were obtained using a sandwich ELISA developed in house (details in the Section 2). Each green point represents an individual chick in a specific time point and group. For each time point and group, red points and blue bars represent the mean ± SEM of 10 individual vaccinated chick measurements and 3 naïve chicks (15 measurements for vaccinated and 6 for naïve, at 36 DOA). Due to heterogeneity of variance between days, the significance of differences between days’ mean values was determined via non-parametric Wilcoxon each paired test; days headed by different letter combinations are significantly different (*p* < 0.05). The significance of differences between groups’ mean values within days was determined via Welch-*t*-test (*p* < 0.05); differences were marked using asterisks *.

As shown in Figure 3 (lower panel), immune complexes were not detected in naïve chicks, while in vaccinated chicks (upper panel), a very low level of IgY-*IBDV* immune complexes was detected as early as 4 DOA and persisted till 14 DOA, whereupon on 18 DOA, the level of immune complexes increased in both levels and responding chicks. Thereafter, on average, the level of immune complexes increased moderately till 32 DOA. A dramatic decline in immune complex levels in all examined chicks was observed at 36 DOA. As in previous observations concerning IBDV viral load in the bursa, high variability between individuals was observed between 18 and 32 DOA. Thus, local immune complexes in the bursa interstitium could represent the impediment of B lymphoblast infection by the vaccine virus, as achieved via MDA.

The *MB-1* vaccine virus migration and localization in the spleen and not in the bursa at the first 14 DOA (Figure 2C) led us to explore whether different levels of maternal antibodies were present in the bursa and the spleen of naïve chicks. Levels of anti-*IBDV* IgY and IgA were determined using an in-house ELISA. 

**Figure 4:**
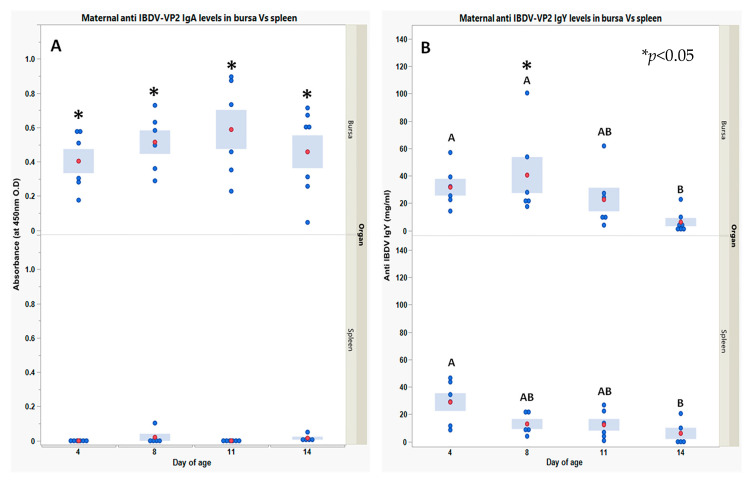
Maternal anti-*IBDV* IgA (**A**) and IgY (**B**) within bursa vs. spleen interstitial fluids in naïve chicks for 14 days post-hatch. IgY and IgA levels were obtained using indirect (quantitative/qualitative, respectively) ELISA developed in house to recognize VP2 of *IBDV* (details in the Section 2). Each blue point represents an individual chick at a specific time point and organ. For each time point and organ, red points and blue bars represent the mean ± SEM of 6 individual chick measurements. For both anti-*IBDV* IgY and IgA, a one-way ANOVA model was used to determine the significance of differences between days’ mean values in the same organ; days headed by different letter combinations are significantly different by Tukey HSD (*p* < 0.05). Due to the heterogeneity of variance between organs within days, the significance of differences between organ mean values was determined via Welch-*t*-test (*p* < 0.05); differences were marked using asterisks *.

Anti-*IBDV* IgA levels (Figure 4A) were significantly higher in the bursa than in the spleen at all ages tested (4 DOA–14 DOA) (mean levels of four-time points in the bursa compared to the spleen). Anti-*IBDV* IgY levels (Figure 4B) were observed in both the spleen and bursa (4 DOA). The levels of anti-*IBDV* IgY persisted longer in the bursa than in the spleen (compare levels on 8 and 11 DOA; insignificant at 11 DOA); however, by 14 DOA, anti-*IBDV* IgY levels had declined to the same levels in both organs.

As the above observations suggested that the spleen is the main organ for *MB-1* vaccine virus location during effective maternal immune protection in the bursa, the next step was to characterize the splenocyte cell populations infected by the virus. For this purpose, FACS and confocal microscopy were conducted on six single-cell suspension pools prepared from spleens from nine vaccinated or nine naïve chicks. The spleens were harvested at 4 DOA (3 days post-*MB-1* vaccination). The cells were stained as described in the Methods section, and each pool was examined using fluorescence confocal microscopy.

**Figure 5:**
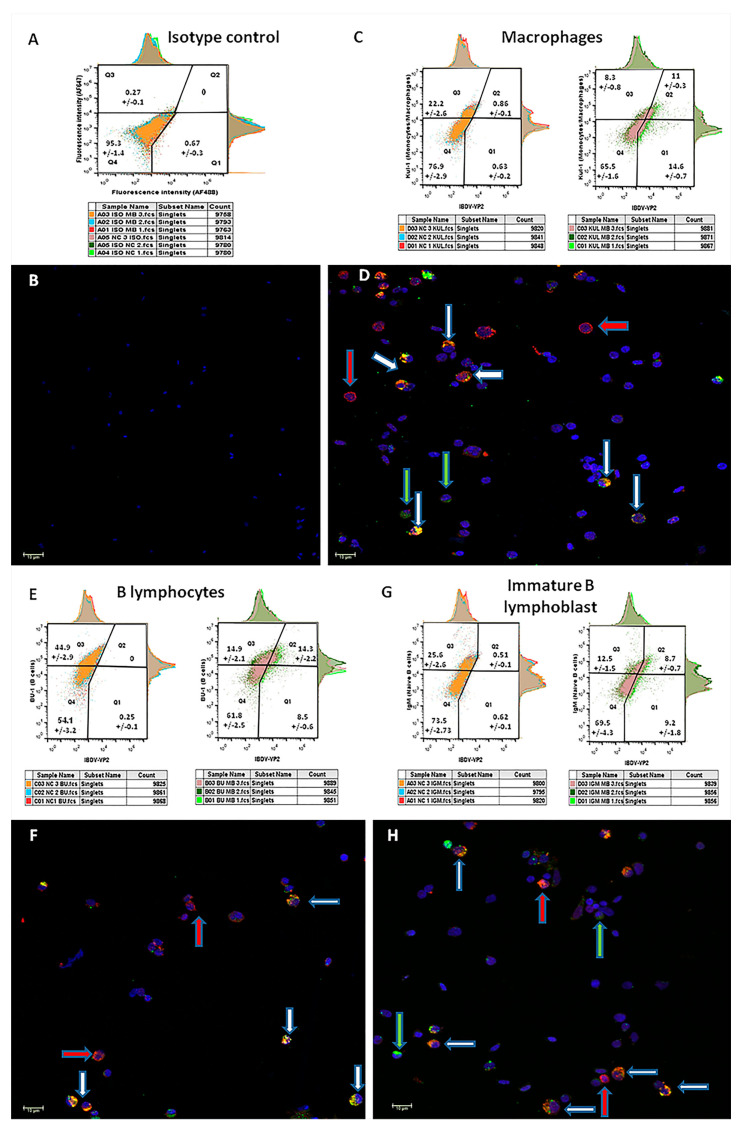
*MB-1* vaccine virus localization in splenocytes at 4 DOA. Splenocyte single-cell suspension pools were made out of nine naïve and nine 1 DOA *MB-1* vaccinated chick’s spleens harvested at 4 DOA (each pool consists of 3 individual spleens of the same treatment). Each pool went through double staining using AF488 monoclonal mouse anti-*IBDV*-VP2 antibody and AF747 antibody against cellular marker for macrophages (**C**) or B-lymphocytes (**E**) or immature B lymphoblasts (**G**)) and 10,000 cells/pool were analyzed. Gating was performed using isotype-negative control (**A**). Mean ± SEM presented in each quarter in the FACS graphs are made of 3 pools (30,000 cells). Following FACS analysis, splenocytes from each pool (only vaccinated pools are presented) were further examined under a fluorescence confocal microscope. Panel B, an isotype negative control, indicates cells were specifically labeled with no false positive background. Four possible populations of cells are visible: double-negative cells only stained with nucleus blue stain (DAPI), *IBDV*-positive and cellular marker-negative-stained with blue and green stains (green arrow), *IBDV*-negative and cellular-marker-positive stained with blue and red stains (red arrow) and double-positive (*IBDV* and cellular marker positive) stained with blue, red and green stains (white arrow).

Double staining with the isotype-negative control (Figure 5, panel A) shows a single splenocyte population located almost exclusively in the fourth quarter in the FACS diagram (95.3+/−1.4%). Accordingly, only DAPI nucleus-stained cells are visible in the paired micrograph (Figure 5, panel B). As expected, no viral signal was detected in the naïve pools. Double staining of samples from vaccinated birds with the *IBDV* marker and the macrophages marker KUL-1 (Figure 5, panel C) revealed that 57.02 ± 3.14% of the macrophages were *IBDV*-positive (Q2/Q2 + Q3), while 43 ± 0.8% of the *IBDV*-infected cells were macrophages (Q2/Q2 + Q1). Double staining with the *IBDV* marker and the B lymphocyte marker BU-1 (Figure 5, panel E) revealed that 48.9 ± 6.94% of the B lymphocytes were *IBDV*-positive, while 62.3 ± 2.5% of the *IBDV*-infected cells were B lymphocytes. Interestingly, double staining of vaccinated samples with the *IBDV* marker and the immature B lymphocyte marker IgM (Figure 5, panel G) revealed a similar proportion of immature B cells (compared to B cells) out of total *IBDV*-infected cells (49.3 ± 2.9%), suggesting that ~85% of the infected B lymphocytes were immature. In all the microscope images taken from *MB-1* vaccinated, double stained samples (Figure 5: panel D—macrophages, Figure 5: panel F—B lymphocytes and panel H—immature B lymphoblasts), four populations of cells are visible: double-negative cells (stained with DAPI only), *IBDV*-positive and cellular-marker-negative (blue and green stains), *IBDV*-negative and cellular-marker-positive (blue and red stains) and double-positive (*IBDV* and cellular marker positive with blue, red and green stains). Moreover, z-stacking demonstrated that cellular markers are limited to cell membranes, while the *IBDV* marker is mostly found in the cell’s cytoplasm. 

Finally, to explore whether the *MB-1* vaccine also elicited mucosal immunity, we measured bile anti-*IBDV* IgA during 18–36 DOA.

**Figure 6:**
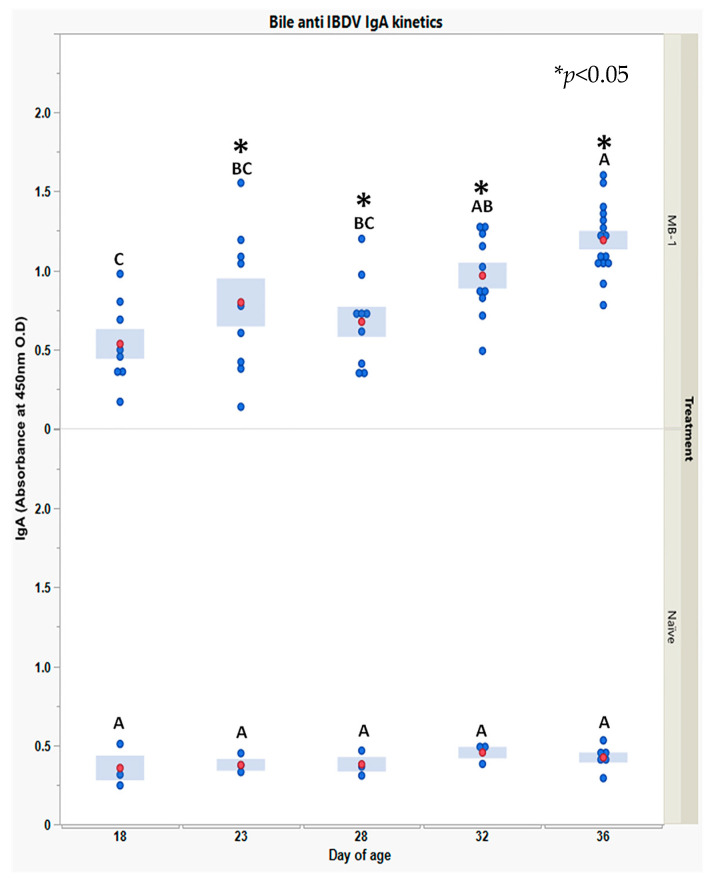
Bile anti-*IBDV* IgA levels in naïve and vaccinated chick kinetics along 18–36 days post-hatch. IgA levels were obtained using indirect qualitative ELISA developed in house to recognize VP2 of *IBDV* (details in the Section 2). Each blue point represents an individual chick at a specific time point and group. For each time point and group, red points and blue bars represent the mean ± 10 individual vaccinated chick’s measurements and 3 naïve chicks (15 measurements for vaccinated and 6 for naïve, at 36 DOA). A one-way ANOVA model was used to determine the significance of differences between days’ mean values in the same group; days headed by different letter combinations are significantly different by Tukey HSD (*p* < 0.05). Due to heterogeneity of variance between groups within days, the significance of differences between groups’ mean values was determined via Welch-*t*-test (*p* < 0.05); differences were marked using asterisks *.

Anti-*IBDV* IgA levels, shown in Figure 6, were very low and stable in the naïve chicks, whereas in the vaccinated chicks, levels were positive on 18 DOA (3 responding chicks) and increased thereafter till 36 DOA, a time at which all chicks examined had secreted anti-*IBDV* IgA into their bile. 

## 4. Discussion

*De novo* antibody production is usually the successful outcome of effective vaccination. The *MB-1* vaccine is different from other *IBDV* live vaccines in that it exhibits two intriguing characteristics: first, the time course of seroconversion is dependent on individual anti-*IBDV* MDA levels, and second, the virus’ survival ability despite maternal protection, eliciting delayed replication following individual immunization. Our current observations in which chicks with low initial MDA underwent early seroconversion while those with high initial MDA underwent later seroconversion are in accordance with previous studies [11]. The virus’ ability to survive maternal protection and to further elicit *de novo* antibody response and immune protection against *IBDV* can be only partially explained by MDA levels, as eventually, chicks with high MDA levels also produced an effective immune response [10,11,12]. As the primary adaptive immune response requires cell proliferation and differentiation, and seroconversion occurred between 14 and 23 DOA, we focused on the target organs for three weeks post-hatching to obtain insights into the *MB-1* immunization process.

As the Bursa of Fabricius is considered the primary target organ for *IBDV* [2,3,22], we were prompted to evaluate the kinetics of viral load in the bursa and other organs using qPCR as in previous studies [9,11,23,24]. Surprisingly, 14 DOA was the earliest time point in which *MB*-1 was detectable in the bursa. The high variability in viral load observed between 18 and 28 DOA could be explained mainly by the different anti-*IBDV* MDA levels between chicks and later on (~24 DOA) along with the appearance of *de novo* antibodies. Viral load increase, as observed from 18 DOA, indicates that the vaccine virus survived by residing in different sites rather than solely in the bursa and rerouted back to the bursa when maternal immunity decreased. Finally, the viral load decrease seen at 36 DOA may suggest *de novo* immune neutralization. 

As *IBDV* is known for its high tropism to immature B lymphoblasts (sIgM^+^) and its ability to reside inside macrophages [1,2,5,7,25], viral load kinetics was examined during 36 days post-vaccination in primary and secondary target organs of *IBDV*, i.e., bursa, blood, thymus, and spleen [2,8,26]. Viral load kinetics in the thymus differed from that in the bursa: the vaccine virus was first detected at 23 DOA (~9 days later than in the bursa), and a relatively low viral load was observed. Moreover, no virus was detected in the blood. These results could be explained by the very low amount of immature B lymphoblasts (3% of lymphocytes) in the thymus [27] and even less so in the peripheral blood [28]. Interestingly, the *MB-1* virus was detected in the spleen as early as 4 DOA, increased slightly till 23 DOA, and finally reached a more significant level at 32–36 DOA. This observation suggested the spleen to be the “alternative” target organ during the time frame with pronounced maternal immunity. 

Since host–pathogen interaction around the primary target organ could greatly contribute to understanding the immunization dynamics [2,7,29,30,31], *IBDV*-IgY immune complex levels in bursa interstitial fluids were determined via ELISA. Very low levels of immune complexes were observed until 14 DOA, while higher levels of immune complexes were observed between 18 and 36 DOA. These observations correlated with the viral load kinetics in the spleen and bursa. We can assume that the viral load in the spleen during the first 14 days was too low to infect bursal cells, being neutralized by maternal antibodies. Once maternally derived antibody levels declined, the *MB*-1 virus could infect and replicate in the bursa cells, generating a high viral load and increasing the *IBDV*-IgY complexes levels. Finally, the sharp decline in the immune complexes levels at 36 DOA along with the decline seen in the spleen and bursa viral load is attributed to the *de novo* antibody immune response, eradicating the virus. Interestingly, immune complexes were not observed in blood from 4 to 14 DOA (data not shown).

The effectiveness of maternal protection is an important factor in the virus’s ability to infect organs. Moreover, high levels of anti-*IBDV* IgA and IgY in the bursa testify to higher levels of maternal protection in the bursa than in the spleen. This observation could be explained by the fact that the bursa is a mucosal organ and is protected by the designated mucosal immunity [32,33]. Additionally, the bursa is anatomically connected to the digestive system via the bursal duct, giving access to unabsorbed and intact maternal IgY derived from the yolk sac and washed down the digestive tract to the bursa to allow neutralization of pathogens [34,35]. Previous studies demonstrating that MDA could neutralize any *IBDV* vaccine applied orally before 10–17 DOA support the present observations on maternal protection and pathogen neutralization around the digestive tract [10,11].

The establishment of the spleen as the main alternative site for the *MB-1* vaccine virus during the first two weeks post-hatching led us to characterize the different infected splenocyte sub-populations. Four DOA (3 days post-*MB-1* vaccination) was selected as the ultimate time point as it represented the earliest time point with both detectable viral load in the spleen and the highest MDA levels. Flow cytometry supported by confocal microscopy showed that the cellular population infected by *MB-1* is essentially macrophages; a higher percentage of macrophages was *IBDV*-positive (~60%) along with intensified fluorescent signal per cell and less so B lymphoblasts. Given the virus’s ability to survive and disseminate within macrophages [5,8], this observation is quite reasonable. In birds, the spleen, the main analog to mammalian lymph nodes, constitutes the compartmentalized microenvironment in which the probability of antigen-macrophage and lymphocyte interaction is increased (antigen processing and presentation) for the initiation of the adaptive immune response. The spleen is also the main organ for homing macrophages after phagocytosis [36,37,38,39]. The results highly suggest that *IBDV-positive* macrophages in the spleen originate from the skin tissue: following vaccine administration by injection, macrophages uptake the vaccine virus and migrate to the spleen. Nevertheless, the ability of the virus to remain viable in macrophages is a key concept in the *MB-1* mode of action, ensuring its long-term viability while maternal immunity is effective.

Finally, the ability of the *MB-1* vaccine to elicit both mucosal and humoral immunity is crucial; mucosal immunity (IgA) provides the first line of protection, avoiding infection, while humoral immunity (neutralizing IgY) provides the second line of protection, limiting the time of infection and therefore, improving prognosis [1,3,13]. 

## 5. Conclusions

This study led us to propose a clear *MB-1* immunization mechanism of action: Following its injection, the *MB-1* vaccine virus carried by skin resident macrophages migrates via the bloodstream to invade the spleen to infect and survive in additional splenic macrophages. The decay in MDA allows the spread, invasion, and replication of the *MB-1* vaccine virus in its target organs. Finally, *de novo* production of anti-*IBDV* IgY provides full immunization and eliminates the *IBD* virus. This mode of action provides a clear explanation of the ability of the *MB-1* virus vaccine to survive in maternally immune chicks and replicate properly to allow efficient, early, and continuous immunization against Gumboro disease.

## Data Availability

Not applicable.
